# Isolation and characterization of Chitosan from shrimp shell waste and the sustainable preparation of salicylic acid-loaded Chitosan nanoparticles for antibiofilm applications

**DOI:** 10.1038/s41598-025-03355-3

**Published:** 2025-06-02

**Authors:** Habiba A. Ahmed, Yousra A. El-Maradny, Manal A. Shalaby, Hany El-Menshawy, Abeer E. Abd EL-Wahab

**Affiliations:** 1 Plant Biochemistry Department, National Research Centre, Dokki, 12622 Giza Egypt; 2 Medical Biotechnology Department, Institute of Genetic Engineering and Biotechnology, City of Scientific Research and Technological Applications (SRTA-City), New Borg EL-Arab, 21934 Alexandria Egypt; 3 Pharmaceutical and Fermentation Industries Development Center, City of Scientific Research and Technological Applications (SRTA-City), New Borg EL-Arab, 21934 Alexandria Egypt; 4 SummerMoon for Food Industries, Alexandria, Egypt

**Keywords:** Eco-friendly, Sustainability, Polymer, Nano formulation, Microbial resistance, And biofilm, Biotechnology, Drug discovery, Microbiology

## Abstract

Microbial biofilms present a significant global health challenge, as they are associated with severe chronic infections and the emergence of antibiotic resistance. Currently, only a limited number of clinically available drugs effectively target microbial biofilms. This underscores the urgent need for the development of new sustainable therapeutic strategies to address biofilm-associated infections. Developing a sustainable and biodegradable preparation for eradicating microbial biofilms is critically important. In this study, chitosan was extracted from shrimp shell waste and utilized to prepare salicylic acid-loaded chitosan nanoparticles (NPs) using various synthesis methods. The particle size of the prepared nanoparticles ranged from 287.4 to 226.3 nm, with zeta potential values between + 36.6 and + 41.3 mV, indicating good stability. The nanoparticles demonstrated safety, with half maximal inhibitory concentration (IC_50_) values ranging from 1009 to 1346 µg/mL. The combination of chitosan and salicylic acid exhibited significant antibiofilm activity against *Escherichia coli*, *Klebsiella pneumoniae*, *Staphylococcus aureus*, and *Candida albicans*, with particularly high efficacy against *Candida albicans*, achieving up to 85% biofilm inhibition. While the particle size and antibiofilm activity of the nanoforms showed minimal differences, formulation M4, using sodium alginate, stands out as the most eco-friendly option. This study highlights the potential of bio-sustainable chitosan-based formulations for combating biofilm formation and addressing antimicrobial resistance.

## Introduction

The deaths of microbial resistance are suspected to reach 10 million per year by 2050, far exceeding those caused by cancer^1^. The increased drug resistance observed in microbes within biofilms, compared to their planktonic form, is mainly due to the biofilm matrix^2^. The primary component of biofilms is extracellular polymeric substances (EPS), which protect bacterial cells within the biofilm from dehydration and shield them from immune responses and antimicrobial drugs^3,4^. Bacterial biofilms consist of microbial communities, and their protective matrix structure reduces the effectiveness of antibiotics by up to 1000 times compared to free-floating bacteria, thus facilitating the development of antibiotic resistance^5^. Even when antimicrobial drugs effectively kill cells within biofilms without disrupting the biofilm structure, the dead biofilm can still facilitate the adhesion of other microbial cells and promote biofilm regrowth^6^. Therefore, both the bacterial cells and the biofilm matrix itself should be targeted in treatment strategies. Degrading the biofilm matrix to enhance drug efficacy has been proposed as a new approach. Given the growing threat of bacterial resistance and the impact of biofilms on human health, there is an urgent need for methods that can both kill bacteria and prevent biofilm formation without promoting increased resistance^7^.

Chitosan is one of the most important derivatives of chitin, which is primarily sourced from crustacean shells, such as those of shrimp or crabs, as well as from the cell walls of fungi. This naturally occurring polysaccharide is cationic, highly basic, and mucoadhesive. As a natural biopolymer, chitosan has garnered significant attention due to its biocompatibility, biodegradability, and non-toxicity. It has received U.S. food and drug administration (FDA) approval for use in tissue engineering and drug delivery applications^8^. Chitosan’s unique properties, including its ability to form films, gels, and nanoparticles, make it a versatile material for various innovative solutions^9^. Currently, chitosan is being intensively explored for its applications in medicine, cosmetics, drug delivery, environmental protection, biotechnology, agriculture, food, and non-food industries, such as water treatment, paper, and textiles^10^. Furthermore, chitosan is effective against bacteria, viruses, and fungi^11,12^. Its bactericidal properties stem from the electrostatic interaction between its NH^3+^ groups and the phosphoryl groups present in bacterial cell membrane phospholipids. This interaction results in the formation of pores in the bacterial cell membrane, making it permeable, and ultimately leading to the rupture of bacterial cells^13^.

Chitosan nanoparticles (NPs) are particularly noteworthy because of their enhanced properties, such as increased surface area, improved solubility, and controlled release capabilities. Synthesizing these nanoparticles allow for a broad range of applications, enhancing the functional value of chitosan^14^. Chitosan NPs have emerged as a promising biodegradable antimicrobial solution, particularly effective against biofilms. Recognized as GRAS (Generally Recognized as Safe) by the FDA, chitosan NPs are well-positioned for diverse biomedical and industrial uses, highlighting their safety, sustainability, and extensive applicability. Chitosan NPs degrade into harmless substances, rendering them ideal for prolonged medical applications, such as wound healing and medical device coatings. Various methods are employed to prepare these nanoparticles, each with unique advantages that cater to specific applications^15,16^. From literature, the properties of chitosan NPs can vary considerably depending on the preparation methods that are used, and the surface modification techniques that are applied, which can lead to applications in completely different fields^17^. Chitosan NPs fabrication technology has emerged as an effective drug delivery strategy, offering advantages such as controlled release, protection of active components from enzymatic or environmental degradation, and localized retention. Additionally, the fabrication methods for nanoparticles are easily scalable and can be applied to a wide variety of drugs^18^. Using chitosan NPs for antimicrobial and antibiofilm applications represents advanced strategies to overcome the limitations of chitosan. Improved formulation methods now allow for a broader range of drugs and even macromolecules to be effectively delivered^13,19^.

Salicylic acid has shown a wide variety of medical benefits, including anti-inflammatory properties, colorectal cancer prevention, and antibacterial and antifungal effects. As a phenolic molecule with an aromatic ring and hydroxyl group. The primary challenge with the external administration of salicylic acid lies in maintaining a long-lasting and continuous effect, as it is highly susceptible to degradation when exposed to light or extreme temperature fluctuations, leading to a reduction in potency and potential loss of biological activity^20^. Additionally, its poor aqueous solubility limits its bioavailability in free form. Incorporating salicylic acid into chitosan NPs aims to enhance its solubility, stability, and controlled release, improving its therapeutic potential. The combination of chitosan and salicylic acid could represent an intriguing area of study, as it creates an entirely natural-based dressing^21^. We chose chitosan for this study due to its biocompatibility, biodegradability, and ability to enhance drug stability and controlled release. Unlike cellulose and other natural polymers, chitosan also has mucoadhesive and antimicrobial properties. Additionally, it is derived from shrimp shell waste, a major pollutant from the seafood industry. Using chitosan not only offers functional benefits but also supports sustainable waste management, making it a suitable choice for this study^22^.

This study aims to sustainably utilize shrimp shell waste for extracting chitosan and to explore its efficacy in antibiofilm applications after nano-formulation with salicylic acid. Additionally, this research explores the ability of various chitosan and salicylic acid nanoparticles to inhibit biofilm formation, while also studying the cytotoxicity of the prepared nanoparticles. Furthermore, these nanoparticles were characterized using Fourier transform infrared (FT-IR), scanning electron microscopy (SEM), transmission electron microscope (TEM), X-ray spectroscopy (EDX), and zeta sizer to examine their physical and chemical properties.

## Materials and methods

### Preparation of Chitosan from the shrimp shells waste

**Fig. 1:**
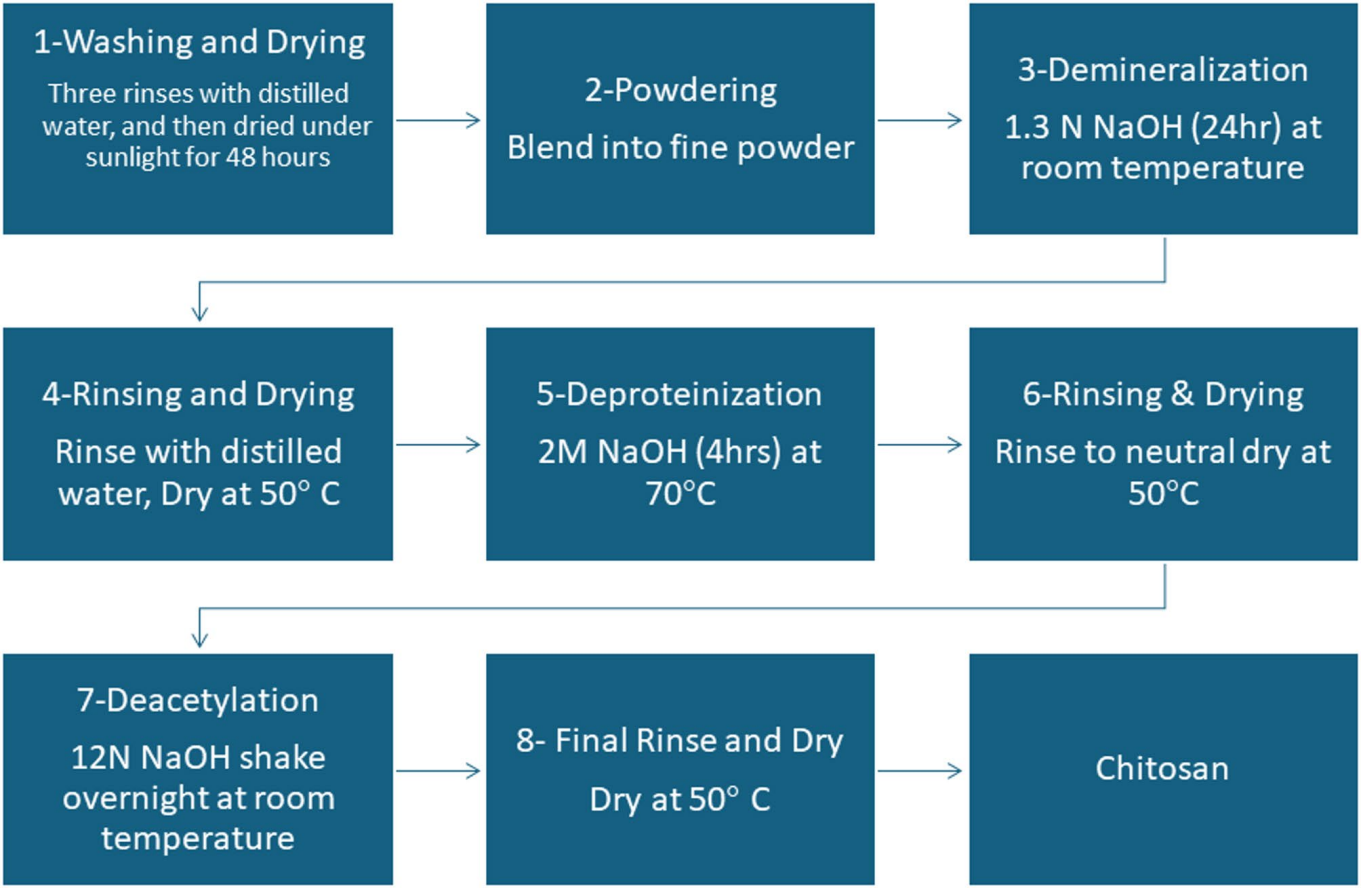
Flow chart illustrating the process of chitosan extraction from shrimps.

Shrimp shell waste was collected from Summer Moon for Food Industries in Alexandria, Egypt. The shells were first washed thoroughly with running water, followed by three rinses with distilled water, and then dried under sunlight for 48 h. Once dried, the shells were blended to produce a uniform, dried powder. The process began with the demineralization of the finely powdered shrimp shells using 1.3 N HCl for 24 h at room temperature. The shells were then rinsed with distilled water to remove any residual acid and dried in an oven at 50 °C. In the next step, deproteinization was carried out by treating the demineralized shells with a 2 N NaOH solution for 4 h at 70 °C. The residue was washed with distilled water to eliminate any remaining NaOH and dried again at 50 °C, yielding chitin. Finally, the chitin was converted into chitosan through deacetylation by immersing it in a 12 N sodium hydroxide solution and shaking it at room temperature. After thorough rinsing with distilled water and drying at 50 °C, the deacetylated chitin—now referred to as chitosan—was ready for use^23^. A detailed illustration of the chitosan extraction method is shown in Fig. 1.

### Yield of Chitosan

The chitosan yield was determined as: (Mass of extracted chitosan [dry powder] / Mass of shrimp shell waste) × 100. This ratio reflects the extraction efficiency by relating the final chitosan mass to the initial waste mass^24^.

### Synthesis of Chitosan NPs loaded with Salicylic acid

**Fig. 2:**
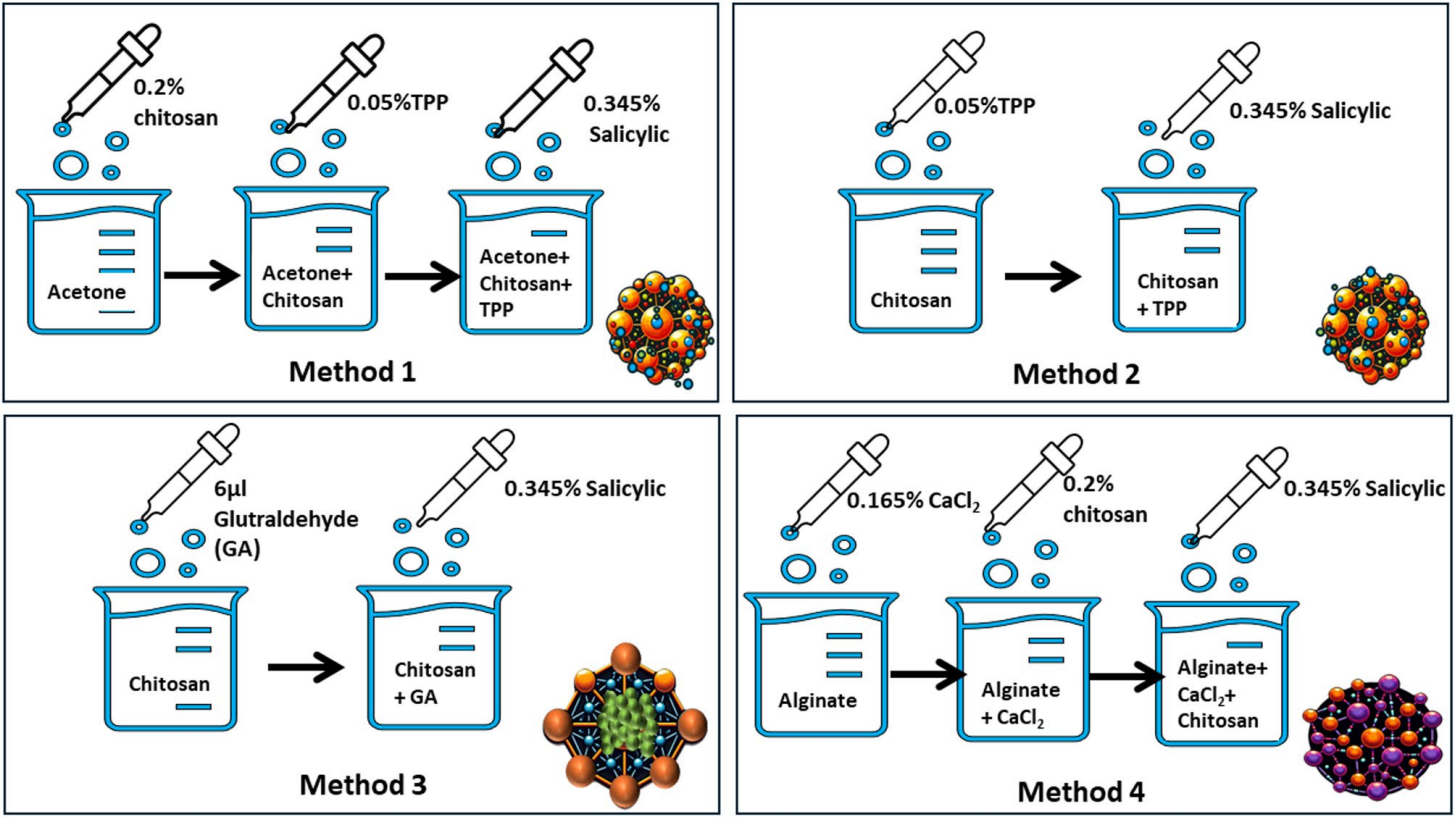
An illustration of the different chitosan NPs preparation methods and the expected crosslinking of each method.

Four attempted methods were used to synthesize chitosan NPs: The TPP method (M1) involves a desolvation-precipitation approach using acetone (dissolution/precipitation), TPP method (M2) acetone-free, the glutaraldehyde method (M3), and the sodium alginate method (M4). Below is a detailed explanation and comparative diagram of the synthesis methods and expected crosslinking of chitosan NPs loaded with salicylic acid (Fig. 2).

*Method 1 (M1) TPP (Dissolvation/Precipitation) method as described by Bangun et al.*^25^. Chitosan NPs were synthesized by preparing a 0.2% w/v chitosan solution in 1% acetic acid, stirred overnight and filtered using Whatman filter paper No. 1. Separately, 10 mg of tripolyphosphate (TPP) was dissolved in 20 mL of distilled water, and 0.069 g of salicylic acid was dissolved in 20 mL of acetone. A total of 100 mL of acetone was stirred at 400 rpm while the chitosan solution was added dropwise over 6 h, followed by overnight storage at 4 °C. TPP was then added dropwise to this mixture, followed by the dropwise addition of salicylic acid. The resulting mixture was stirred at 600 rpm overnight. Finally, the mixture was centrifuged at 3000 rpm for 15 min to separate the particles, and the supernatant was collected and stored at 4 °C until use^26^.

*Method 2 TPP (M2) as described by Manimekalai et al.*,* 2017*^27^. Following a slightly modified procedure from the TPP method, 50 mL of 0.2% w/v chitosan was dissolved in 1% acetic acid by stirring overnight and then filtered through Whatman filter paper No. 1. TPP (10 mg) was dissolved in 20 mL of distilled water, and 0.069 g of salicylic acid was dissolved in 20 mL of acetone. The chitosan solution was stirred at 400 rpm while TPP was added dropwise, followed by the dropwise addition of salicylic acid. The mixture was maintained at 600 rpm overnight, after which it was centrifuged at 3000 rpm for 15 min. The supernatant was collected and stored at 4 °C until use.

*Method 3 Glutaraldehyde (M3) as reported by Islam et al.*,* 2019*^28^, with some modifications: Chitosan NPs were synthesized by initially dissolving chitosan powder in 1 M HCl and stirring for over 24 h to obtain a homogeneous, viscous chitosan solution. A chitosan stock solution was prepared by dissolving chitosan in 1 M HCl. Chitosan NPs were formed by adjusting the pH of the stock solution to 8.0 using NaOH, followed by washing and drying the sample at 50 °C for 24 h. To prepare chitosan-N-glutaraldehyde (Cs-N-GA), the pH of the chitosan stock solution was adjusted to 8.0, and glutaraldehyde (6 µL GA per 5 mL chitosan) was added. The crosslinking reaction between the amine groups of the chitosan polymer and glutaraldehyde formed stable amine bonds. Additionally, 0.069 g of salicylic acid was dissolved in 20 mL of acetone and added dropwise to the mixture.

*Method 4 Sodium alginate (M4*) following *Lertsutthiwong et al.*,* 2009*^29^. In this method, 4 mL of 0.8 mg/mL chitosan was dissolved in 1% acetic acid by stirring overnight. Separately, an alginate solution (2 mg/mL) was dissolved in 10 mL of distilled water, and calcium chloride (3.3 mg/mL) was dissolved in 2 mL of distilled water. Additionally, 0.069 g of salicylic acid was dissolved in 20 mL of acetone. The alginate solution was agitated while Ca-Cl₂ was added dropwise. Subsequently, the chitosan solution was added dropwise to the mixture, followed by the addition of salicylic acid. The resulting mixture was stirred at 600 rpm overnight, followed by centrifugation at 3000 rpm for 15 min. The supernatant was collected and stored at 4 °C until further use.

### Fourier transform infrared spectroscopy (FT-IR)

Spectrum analysis was performed on a freeze-dried sample of chitosan nanocomposite using a Bio-Rad FT-IR-40 (USA). For this, 10 mg of the sample was mixed with 100 mg of dried potassium bromide (KBr) from Merck Chemicals (Darmstadt, Germany) and compressed into a salt disc with a diameter of 10 mm. The spectrum was recorded over a wavenumber range of 4000 to 500 cm⁻¹.

### Transmission Electron microscopy (TEM)

This method was used to investigate the morphology and particle size of the synthesized chitosan nanocomposite. The nanoparticle solution was sonicated for 5 min to improve dispersion before sample preparation. A drop of the sonicated solution was placed on a carbon-coated copper TEM grid (200–300 mesh), allowed to dry at room temperature, and imaged using an FEI TECNAI G2 F20 S-TWIN (Thermo Fisher Scientific, Waltham, MA, USA).

### Scanning Electron microscopy (SEM) analysis

SEM was employed to examine the microstructure of the chitosan. The lyophilized chitosan sample was cut using a punch and affixed to an adhesive carbon stub. Imaging was conducted using a Tabletop SEM (JEOL 6340, Japan) operating at 15 kV.

### Particle size, polydispersity index, and zeta potential analysis

The DLS technique was used to determine the size distribution and zeta potential of the synthesized chitosan NPs. Measurements were carried out on a Malvern Zeta sizer (Malvern Instruments Corp., Malvern, United Kingdom) in solutions of pH = 5. All samples were diluted with Millipore-filtered (MF-Millipore™ Membrane Filters) deionized water to an appropriate scattering intensity.

### X-ray spectroscopy (EDX) and mapping analysis

For EDX characterization using TEM, a drop of the sonicated nanoparticle suspension is placed on a carbon-coated copper grid, dried at room temperature, and inserted into the TEM chamber. Chemical purity was evaluated using energy-dispersive X-ray spectroscopy (EDX). This technique involves transmitting the sample with an electron beam, which is then detected by a specialized electron microscope. The resulting data was subsequently analysed using an EDX analyser.

### Cytotoxicity assessment

The cell viability of salicylic acid and chitosan NPs with and without salicylic acid was evaluated following the Mosmann method^30^, using human skin fibroblast (HSF) cells. HSF cells were sourced from the American Type Culture Collection (ATCC) and cultured in Dulbecco’s Modified Eagle Medium (DMEM) high glucose and supplemented with 5% fetal bovine serum (FBS) (Lonza, USA) and 1%penicillin-streptomycin solution (100 IU/mL penicillin and 100 mg/mL streptomycin, Sigma-Aldrich, USA). These cells were seeded into 96-well plates at a density of 5 × 10^3^ cells per well. Next, various concentrations (1000, 500, 250, 125, 62.25, and 31.5 µg/mL) of chitosan NPs, salicylic acid, and chitosan NPs loaded with salicylic acid were incubated with HSF cells in a 5% CO₂ incubator at 37 °C for 48 h. After incubation, a solution of MTT (0.5 mg/mL in PBS) was added to each well, followed by a 4-hour incubation under the same conditions, and 200 µL dimethyl sulfoxide (DMSO) was subsequently added. The absorbance at 570 nm was measured using an ELISA reader (BMG LabTech, Germany) to determine the percentage of cell viability. The safe concentration, defined as EC100 (100% cell viability), was calculated using GraphPad Instat 8 software. Moreover, the morphological changes in HSF cells treated with different concentrations of chitosan were compared with untreated control cells using phase-contrast microscopy (Olympus, Germany).

### Anti-biofilm assay

The anti-biofilm activity was evaluated both qualitatively, using light microscopy, and quantitatively, by measuring the absorbance of the crystal violet staining. Luria Bertani (LB) broth was inoculated with four microorganisms and incubated separately for 24 h in a shaking incubator. The microorganisms included two Gram-negative bacteria, *Escherichia coli* ATCC 25,922 and *Klebsiella pneumoniae* ATCC 13,883, one Gram-positive bacterium, *Staphylococcus aureus* ATCC 25,923, and one yeast, *Candida albicans* ATCC 10,231, were obtained from ATCC (American Type Culture Collection) (Manassas, VA, USA). A bacterial count equivalent to 0.5 McFarland (10⁶ CFU/mL) was prepared in trypticase soy broth supplemented with 0.2% glucose, and 200 µL of the suspension was added to a 96-well flat-bottom culture plate (in triplicates). The plates were incubated overnight at 37 °C for 48 h. Positive controls consisted of culture without the addition of tested compounds, while negative controls contained broth without microorganism. After incubation, the well contents were removed, and the plates were washed three times with sterile phosphate-buffered saline (PBS) to eliminate planktonic microorganisms. Fresh Mueller-Hinton broth (200 µL) was added to each well along with varying concentrations of the tested compounds, and the plates were re-incubated at 37 °C for 24 h.

Following the second incubation, the plates were washed three times with PBS and left to dry before being stained with 1% crystal violet for 30 min. Excess stain was removed by washing with sterile distilled water, and the plates were then left to air-dry overnight. For qualitative assays, the plates were examined under phase-contrast microscopy (Olympus, Germany) at 40× magnification, and the results were compared to the positive control, which contained untreated organisms, and the negative control, which contained media without microorganisms or tested compounds. To quantify the results, the biofilms were solubilized using 100 µL of 30% glacial acetic acid, and absorbance was measured at 590 nm with a microplate reader. Higher optical density values indicated a greater number of cells absorbing the dye^31^. The percentage of biofilm inhibition was calculated using the following formula:

$$\begin{aligned} & Biofilm\;inhibition\;\left( \% \right)=\left( {\left[ {{\text{Control}}\;{\text{O}}{{\text{D}}_{{\text{59}}0}} - {\text{Treated}}\;{\text{O}}{{\text{D}}_{{\text{59}}0}}} \right]/{\text{Control}}\;{\text{O}}{{\text{D}}_{{\text{59}}0}}} \right) \times {\text{1}}00 \\ & Biofilm\;fold\;reduction={\text{Control}}\;{\text{O}}{{\text{D}}_{{\text{59}}0}}/{\text{Treated}}\;{\text{O}}{{\text{D}}_{{\text{59}}0}} \\ \end{aligned} $$

### Statistical analysis

The statistical analyses were conducted in triplicate (*n* = 3), and GraphPad Prism Instat 8 software. was employed to assess the significance of the results using one-way analysis of variance (ANOVA) at a probability level of *p* < 0.05. The various parameter values are presented as the mean ± standard deviation (SD). Furthermore, the IC50 values were calculated through nonlinear regression modelling using GraphPad Prism Instat 8 software.

## Results and discussion

### Yield of Chitosan

The yield of chitosan extracted from shrimps was (28 g / 120 g) × 100 = 23.33%. This result indicates that 23.33% of the original shrimp shell waste was successfully converted into dry chitosan powder. The obtained yield falls within the commonly reported range of 5.6–30% found in previous studies. For instance, studies by Nanzin et al. reported chitosan yields between 7.5% and 18% using similar acid and alkaline concentrations, while other studies by Nouri et al. 2016 achieved yields as high as 19.74 using more concentrated alkaline solutions. These results suggested that the extraction method used was effective and consistent with established procedures^32–34^.

### FT-IR spectroscopy

**Fig. 3:**
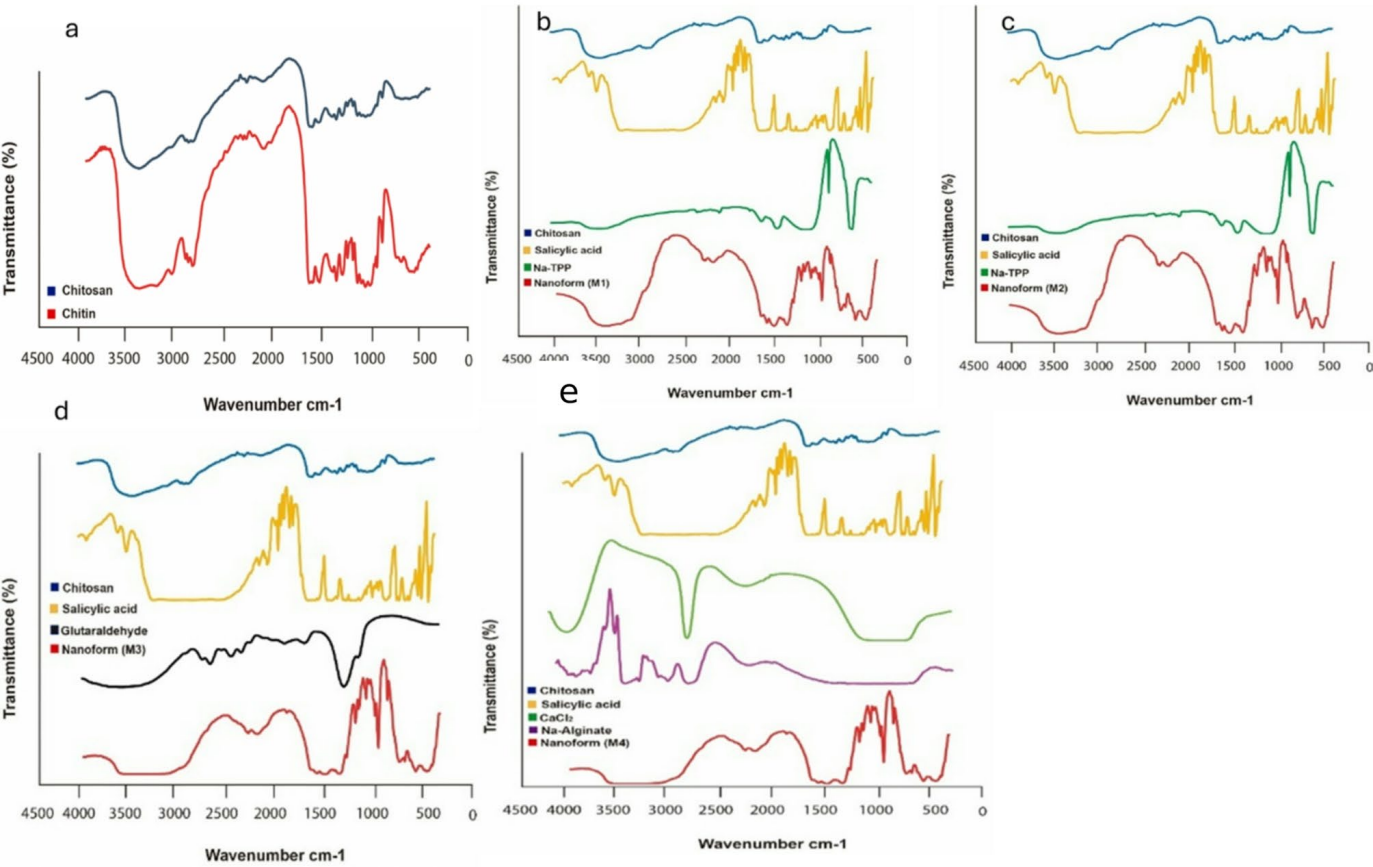
Fourier-transform infrared spectroscopy (FT-IR) spectra of chitin and chitosan (**a**), M1 (**b**), M2 (**c**), M3 (**d**) and M4 (**e**).

The FT-IR spectra of chitin and chitosan exhibited slight differences, primarily manifested as shifts in their bands **(**Fig. 3a**).** These shifts were attributed to the loss of acetyl groups in chitosan. Chitin and chitosan exhibited absorption peaks at 3435 cm⁻¹ and 3443 cm⁻¹, respectively. These peaks corresponded to the stretching vibrations of -OH and amine N-H symmetric vibrations. Interestingly, sharp absorption around 3500 cm^− 1^, indicative of free OH groups, was absent in all samples. The bands observed at 1072 cm^− 1^ (chitin) and 1071 cm^− 1^ (chitosan) were attributed to the stretching vibrations of -C-O groups, with chitosan exhibiting depressed C-O groups due to deacetylation. Additionally, the absorption band at 1413 cm^− 1^ in chitosan characterized the stretching vibration of the amino group. Further characteristic peaks between 1072 –1025 cm^− 1^ and 530–572 cm^− 1^ confirmed the saccharide structure of chitin and chitosan, respectively, owing to variations in the C-O groups^35^. Figure 3 (b and c) displays the FT-IR spectra of the first and second forms of chitosan-loaded salicylic acid nanoparticles, utilizing TPP as a linker. A prominent peak at 3450 cm^− 1^ corresponds to N-H and O-H stretching, as well as intramolecular hydrogen bonds. This peak is notably sharper in the Cs-NPs, indicating enhanced hydrogen bonding^36^. The peaks observed around 2923 cm^− 1^ and 2883 cm^− 1^ are attributed to C-H symmetric and asymmetric stretching, respectively. Additionally, the presence of residual N-acetyl groups is confirmed by peaks around 1629 cm^− 1^ (C = O stretching of amide I) and 1561 cm^− 1^ (NH2 groups). Interestingly, these peaks shift to 1636 cm^− 1^ (M1), 1637 cm^− 1^ (M2), and 1555 cm^− 1^ (M1), 1556 cm^− 1^ (M2) in the FT-IR spectra of salicylic acid-chitosan-NPs, indicating interaction between NH^3+^ groups of chitosan and phosphate groups of TPP^36,37^. This interaction is further supported by the reduced intensity of the amide (1637 cm^− 1^) peak in salicylic acid-chitosan-NPs compared to pure chitosan. The presence of CH2 bending and CH3 symmetrical deformations is confirmed by bands observed around 1410 cm^− 1^ and 1337 cm^− 1^, respectively^38^. Moreover, the FT-IR spectrum of salicylic acid-chitosan-NPs exhibits characteristic peaks of TPP at around 1214 cm^− 1^ (stretching vibration of P = O), 1144 cm^− 1^ (symmetric stretching vibrations in the O − P = O group), and at around 805 cm^− 1^ (asymmetric stretching vibration of the P − O−P bridge)^38,39^. Figure 3(d) shows the FT-IR spectrum of nano formulation 3 using glutaraldehyde as a linker, illustrating subtle changes in the surface-modified nanocarrier and nano-formulations. In salicylic acid-chitosan-NPs, distinct peaks were observed with shifts at 2316, 1552, 1051, and 641 cm-1. Furthermore, new peaks appeared at 2236, 1943, and 471 cm^− 1^, which strongly indicates surface modification compared to the chitosan control. These spectral changes are likely attributed to the presence of loaded salicylic acid within the formulation. Figure 3(e) presents the FT-IR spectrum of nano formulation 4, illustrating overlapping peaks that suggest interactions among chitosan, sodium alginate, and salicylic acid. The spectrum reveals the presence of peaks from each component within the nano formulation, indicative of their combined presence and potential interactions. New peaks appearing at 1961 and 1732 cm^− 1^ imply chemical reactions involving salicylic acid with other ingredients in the formulation. Key absorption bands include a prominent peak at 3396 cm^− 1^, attributed to the amine and hydroxyl groups present in both chitosan and alginate. The presence of a peak at 1067 cm^− 1^ indicates the presence of amino groups in chitosan. Furthermore, the peak at 1622.38 cm^− 1^ suggests an interaction involving the carboxylate group of chitosan with alginate interaction. These spectral features provide valuable insights into the complex chemical interactions occurring within nano formulation 4, highlighting the interplay among its components^40,41^. M1 in Fig. 3b, shows different peak intensities compared to M2 in Fig. 3c, suggesting variations in crosslinking or molecular interactions. Certain peaks in Fig. 3c, shifted slightly compared to Fig. 3b, indicating different degrees of interaction between salicylic acid, Na-TPP, and chitosan. Additional peaks in Fig. 3c: M2 exhibits additional peaks and broadening in the amide (1600–1700 cm⁻¹) and phosphate regions (~ 1200 cm⁻¹), suggesting a stronger interaction or a different structural arrangement compared to M1 in Fig. 3b and M2 Fig. 3c exhibits greater peak shifts, broadening, and more integrated peaks, suggests that M2 is more stable and uniform than M1. M1 Fig. 3b, has more distinct and separate peaks, indicating weaker interactions and less stability.

### Transmission Electron microscope TEM analysis

**Fig. 4:**
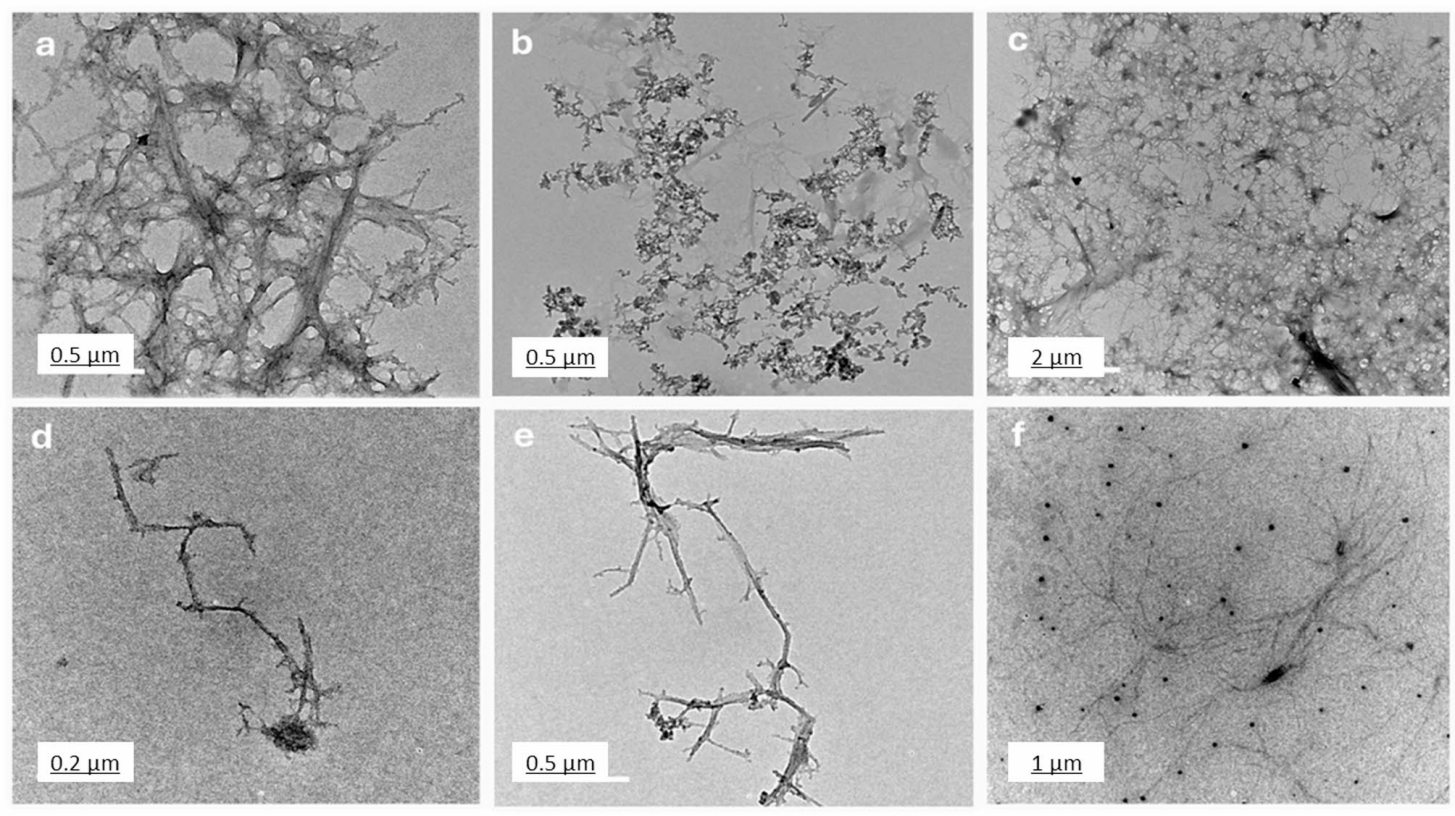
TEM images of chitosan (**a**), salicylic (**b**), salicylic-loaded chitosan NPs (M1 (**c**), M2 (**d**), M3 (**e**) and M4 (**f**)).

Figure 4 presents TEM images (a-f), highlighting the morphological differences among chitosan, salicylic acid, and salicylic acid-loaded chitosan NPs prepared using various methods. Image (Fig. 4a) illustrates a dense fibrous network of chitosan, likely formed through extensive cross-linking. Figures (4b) depicts discrete salicylic acid particles with minimal aggregation, indicative of a preparation method that limits particle interaction. Images (Fig. 4c) through (Fig. 4f) represent salicylic acid-loaded chitosan NPs synthesized using four distinct methods. M1 (Fig. 4c), involving dissolvation /precipitation, shows clustered aggregates with irregular structures, suggesting slower reaction rates or insufficient stabilization during synthesis^42^. M2 (Fig. 4d), employing the TPP method, produces linear, chain-like nanoparticles resulting from processes that promote linear polymerization or anisotropic growth. M3, the glutaraldehyde method (Fig. 4e) generates branched, tree-like formations, achieved through conditions favoring branched polymer growth, such as varying chitosan and cross-linker concentrations or facilitating self-assembly^43^ Finally, the sodium alginate M4 (Fig. 4f) creates a dispersed distribution of nanoparticles, with effective stabilization techniques like ultrasonication or high shear mixing preventing aggregation. These TEM images emphasize the influence of preparation methods on nanoparticle morphology, yielding diverse structures such as dense networks, clustered aggregates, linear chains, and branched or dispersed formations. Understanding these variations is essential for tailoring chitosan NPs for specific applications^44^. Based on the TEM analysis, the estimated size of the synthesized chitosan NPs varied depending on the cross-linking method across the four methods. The TPP-based method (M1 and M2) resulted in nanoparticles with a small and uniform size ranging from 80 nm to 250 nm. Furthermore, nanoparticles produced using the glutaraldehyde method (M3) and sodium alginate method (M4) formed larger nanoparticles, ranging from 150 nm to 400 nm, with network-like structures. These results confirmed that while TPP leads to more uniform, smaller particles, glutaraldehyde and sodium alginate produce larger, network structures.

The TEM images highlight different chitosan nanoparticle morphologies and their potential applications. Network structures (Image a) are ideal for applications requiring high surface area and porosity, such as drug delivery and tissue engineering. Clustered aggregates (Fig. 4c) may be useful in catalysis or adsorption processes^45^. Linear chains (Fig. 4d) are beneficial for applications needing anisotropic properties, like nanocomposites or tissue scaffolds. Branched structures (Fig. 4e) are advantageous for complex architectures, such as advanced drug delivery systems. Dispersed particles (Fig. 4f) are optimal for applications requiring minimal aggregation, such as coatings or nanofluids. TEM analysis (Fig. 4) revealed the formation of network-like nanostructures in our formulation. While our study did not directly measure drug loading or release kinetics, previous research has shown that such nanostructures can enhance drug loading capacity and promote sustained release^46^. Furthermore, the presence of both networked and dispersed nanoparticles may suggest improved biofilm penetration. Tailoring synthesis methods enables customization of nanoparticle properties for specific functions^47^.

### EDX analysis

**Fig. 5:**
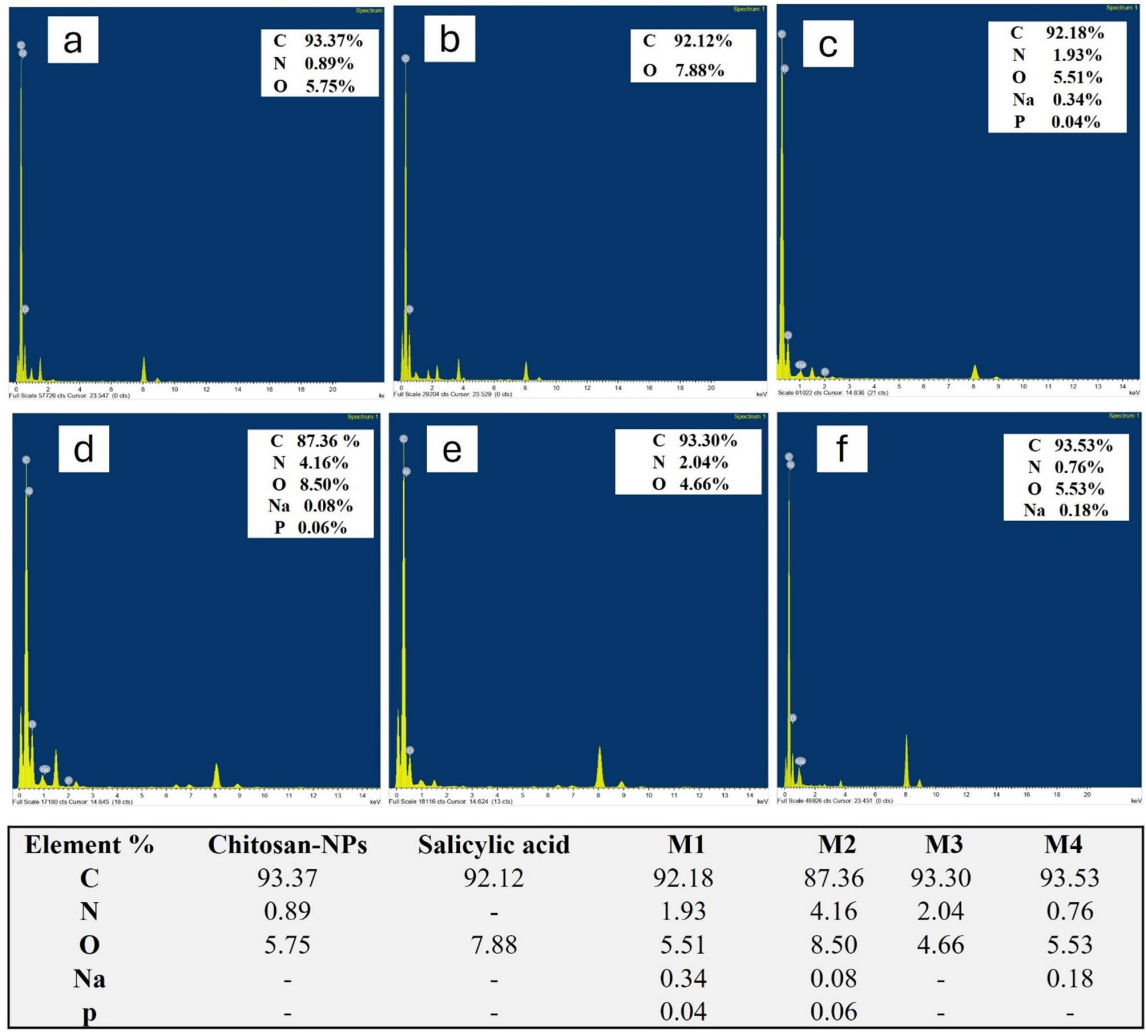
EDX spectra of chitosan (**a**), salicylic (**b**), salicylic-loaded chitosan NPs (M1 (**c**), M2 (**d**), M3 (**e**) and M4 (**f**)).

Figure 5 (a-f) presents the elemental composition of various substances as determined by EDX analysis, providing insight into the chemical makeup of chitosan, salicylic acid, and four different nano formulations. The analysis reveals that chitosan (Fig. 5a) is predominantly composed of carbon (93.37%), with oxygen making up 5.75% and a small amount of nitrogen (0.89%). This elemental composition is consistent with the molecular structure of chitosan, which primarily consists of carbon chains with oxygen and nitrogen functional groups^48^. In contrast, salicylic acid (Fig. 5b) exhibits a composition of 92.12% carbon and 7.88% oxygen, reflecting its organic, crystalline nature where oxygen is a key component of the carboxyl and hydroxyl groups.

M1 (Fig. 5c), which includes chitosan NPs loaded with salicylic acid, shows a slightly different elemental distribution. The carbon content is 92.18%, oxygen is 5.51%, and nitrogen is 1.93%. Additionally, small amounts of sodium (0.34%) and phosphorus (0.04%) are present, likely due to the incorporation of salts or crosslinking agents during the nanoparticle preparation process^49^. M2 (Fig. 5d), which also incorporates chitosan and salicylic acid, reveals 87.36% carbon, 8.50% oxygen, and a higher nitrogen content of 4.16%, indicating the presence of more nitrogen-rich crosslinking agents or additives. Minor traces of sodium (0.08%) and phosphorus (0.06%) are also detected, suggesting that the formulation contains other chemical components that contribute to its structural properties^50^. For M3 (Fig. 5e), the elemental composition shows 93.30% carbon, 4.66% oxygen, and 2.04% nitrogen. This formulation closely resembles the composition of chitosan, with a slight increase in nitrogen, which may be due to the introduction of additional nitrogen-based crosslinking agents or other stabilizers during synthesis. Finally, M4 (Fig. 5f) exhibits a composition of 93.53% carbon, 5.53% oxygen, and 0.76% nitrogen, with small amounts of sodium (0.18%). The higher carbon and oxygen percentages suggest that the formulation is primarily composed of chitosan and its derivatives, with a minimal contribution from other elements. The compositional differences observed in the EDX analysis influence the stability, bioactivity, and interactions of the nanoparticles with microbial cells. The variations in nitrogen content across formulations suggest differences in crosslinking density, which can impact nanoparticle stability. Higher nitrogen levels in M2 and M3 indicate the presence of nitrogen-rich crosslinkers, which enhance structural integrity and influence drug release kinetics^51^. The presence of sodium in M1, M2, and M4 suggests potential ionic interactions that can affect nanoparticle dispersion, preventing agglomeration and surface charge, influencing stability in biological environments^52^. Additionally, phosphorus detected in M1 and M2 may indicate residual crosslinking agents or stabilizers that could alter nanoparticle surface properties, affecting microbial adhesion and interaction. Oxygen content variations suggest differences in hydrophilicity, which can influence solubility, bioavailability, and penetration into microbial biofilms^53^. M2, with the highest oxygen content, may exhibit enhanced water affinity, potentially improving drug release and biofilm penetration. Conversely, formulations with higher carbon content (M3 and M4) suggest a more hydrophobic nature, which could impact cellular uptake and antimicrobial activity^54^. Overall, the EDX analysis provides a detailed understanding of the elemental makeup of the various formulations, indicating the successful incorporation of chitosan and other components like salicylic acid, crosslinking agents, and salts. The variations in nitrogen, sodium, and phosphorus content across the formulations highlight the influence of different synthesis methods and crosslinking agents on the final chemical composition, which plays a crucial role in determining the nanoparticles’ properties and their potential applications^55,56^.

### Morphology of Chitosan NPs (SEM)

**Fig. 6:**
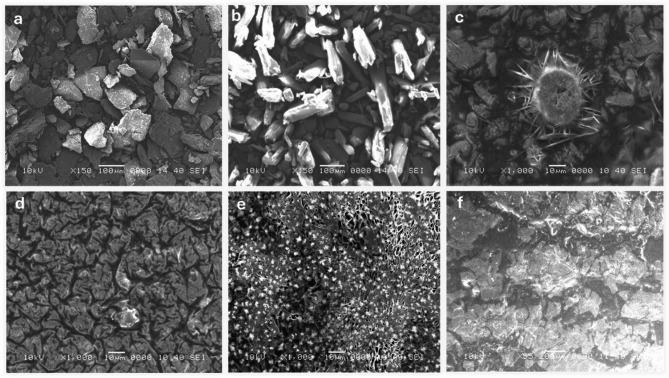
SEM images of chitosan (**a**), salicylic (**b**), salicylic-loaded chitosan NPs (M1 (**c**), M2 (**d**), M3 (**e**), M4 (**f**)).

The SEM images (Fig. 6 (a-f)) reveal the morphological differences in chitosan and its composites prepared by various methods. Bulk chitosan (Fig. 6a) shows an irregular, flaky surface typical for biopolymers in their unmodified form^57^. Salicylic acid (Fig. 6b) exhibits crystalline structures with sharp edges, reflecting its small molecule, crystalline nature. Chitosan NPs prepared by desolvation (Fig. 6c) display a unique star-shaped morphology with needle-like projections. TPP-crosslinked chitosan NPs (Fig. 6d) have a wrinkled surface and a more uniform, densely packed appearance. Glutaraldehyde-prepared nanoparticles (Fig. 6e) show a porous structure, indicating successful crosslinking. Lastly, sodium alginate-prepared nanoparticles (Fig. 6f) are rough and agglomerated, suggesting interactions between chitosan and alginate. Star-shaped nanoparticles (desolvation method) may enhance bacterial membrane disruption due to their high surface area and sharp edges, while wrinkled, densely packed nanoparticles (TPP-crosslinked) offer greater stability and sustained drug release, supporting prolonged antibiofilm action. Porous nanoparticles (glutaraldehyde-crosslinked) allow for higher drug encapsulation and controlled diffusion, improving bacterial eradication, whereas rough, agglomerated nanoparticles (sodium alginate-crosslinked) may affect dispersion and biofilm penetration, potentially influencing antimicrobial efficiency. These morphological variations highlight the influence of different preparation methods and crosslinking agents on nanoparticle formation^58–60^.

The variation in particle size, shape, and surface morphology shows that the choice of crosslinking agent and preparation method significantly affects chitosan NPs’ properties. TPP crosslinking offers uniformity and density, while glutaraldehyde creates porosity, useful for drug delivery. These findings underscore chitosan’s versatility and the importance of selecting the right methods to tailor its properties for specific applications. Future studies could focus on drug loading, release kinetics, and biocompatibility to explore their biomedical potential^61,62^.

### Particle size distribution

**Fig. 7:**
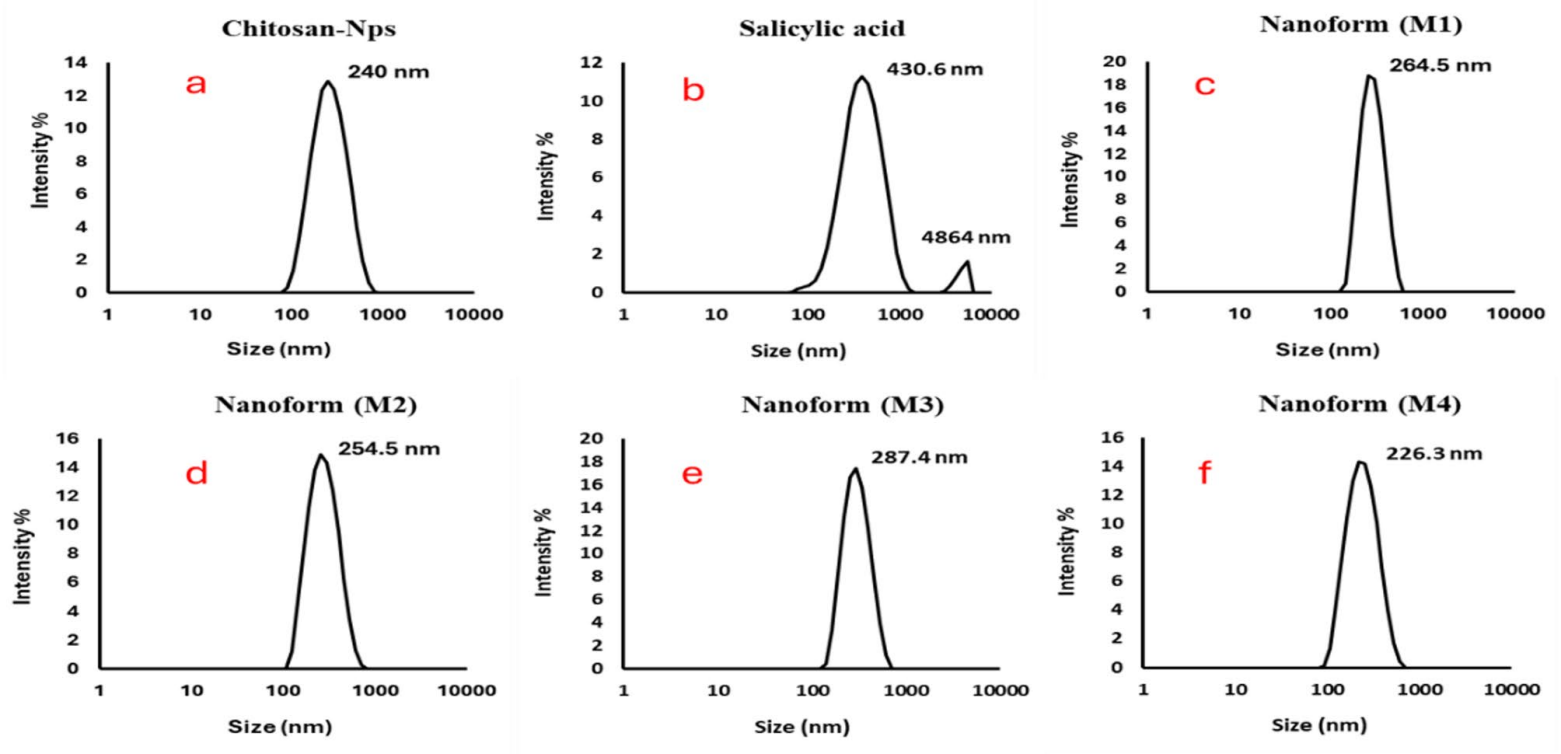
Particle size distribution of chitosan (**a**), salicylic (**b**), salicylic-loaded chitosan NPs (M1 (**c**), M2 (**d**), M3 (**e**) and M4 (**f**)).

Figure 7 (a-f) shows a comprehensive analysis of the size distribution and zeta potential of chitosan, salicylic acid, and salicylic acid-loaded chitosan NPs prepared using various methods. The DLS analysis reveals a narrow size distribution for all the nanoparticle formulations, with mono dispersity indices of 0.139 for chitosan (Fig. 7a), 0.261 for salicylic acid (Fig. 7b), and 0.118, 0.135, and 0.166 for the different salicylic acid-loaded chitosan nanoparticle preparations (M1 (Fig. 7c), M2 (Fig. 7d), M3(Fig. 7e), and M4 (Fig. 7f), respectively). These indices indicate relatively uniform particle sizes, with an average diameter of approximately 254 nm across all samples. The consistency of the particle sizes measured through DLS aligns closely with the particle sizes observed in the TEM images, providing additional confirmation of the accuracy of the nanoparticle measurements^63^.

Furthermore, Table 1 provides the zeta potential measurements that offer crucial insights into the stability and dispersibility of the nanoparticles. Chitosan NPs exhibit a zeta potential value of 47.6 mV, while salicylic acid alone shows a much lower value of -2.3 mV, reflecting its less stable nature. The salicylic acid-loaded chitosan NPs, prepared by the four different methods, show zeta potential values ranging from + 36.6 mV to 41.3 mV for methods 1 to 4, respectively. These positive zeta potential values suggest that the nanoparticles are well-dispersed, with strong electrostatic force between particles, preventing aggregation and ensuring their stability in suspension. The higher positive zeta potential values observed for the salicylic acid-loaded chitosan NPs indicate enhanced dispersion and stability, which is beneficial for their potential use in various applications, including drug delivery systems and biomedical formulations^64^. The data presented in Fig. 4; Table 1 not only highlight the uniformity in size distribution but also emphasize the importance of the preparation method in controlling the nanoparticle characteristics, such as particle size, dispersion, and stability^55,58^. These properties are critical for tailoring the nanoparticles for specific applications, and the results suggest that careful selection of preparation techniques can optimize the functional properties of chitosan NPs for targeted use in fields such as drug delivery, tissue engineering, and nanomedicine^60,63^.

**Table 1:** Polydispersity index and zetapotential of Chitosan, Salicylic, salicylic-loaded Chitosan NPs M1, M2, M3 and M4. Values are presented as mean ± standard deviation.

	Chitosan-Nps	Salicylic-acid	M1	M2	M3	M4
Pdi	0.139 ± 0.00	0.261 ± 0.00	0.118 ± 0.00	0.118 ± 0.00	0.135 ± 0.00	0.166 ± 0.00
Zeta potential	+ 47.6 ± 3.97	− 2.33 ± 0.5	+ 36.6 ± 3.99	+ 38.9 ± 4.76	+ 41.3 ± 4.12	+ 37.5 ± 3.24

### Cell viability assay

**Fig. 8:**
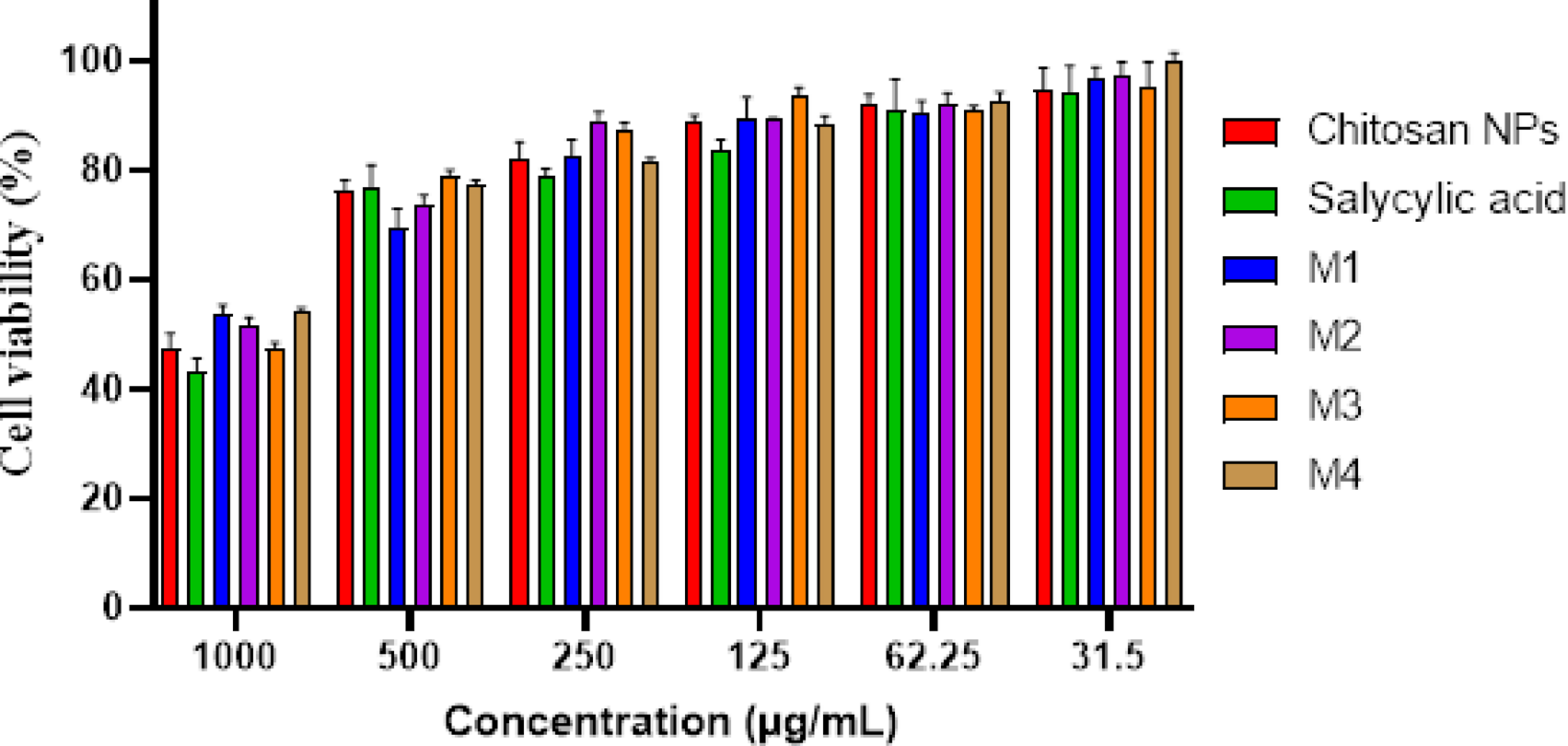
Histogram represents the percentage of viability of human skin fibroblast (HSF) cells treated with chitosan, salicylic, salicylic-loaded chitosan NPs M1, M2, M3 and M4. Data represent mean viability ± standard deviation (SD), (*n* = 3) from MTT assays.

**Fig. 9:**
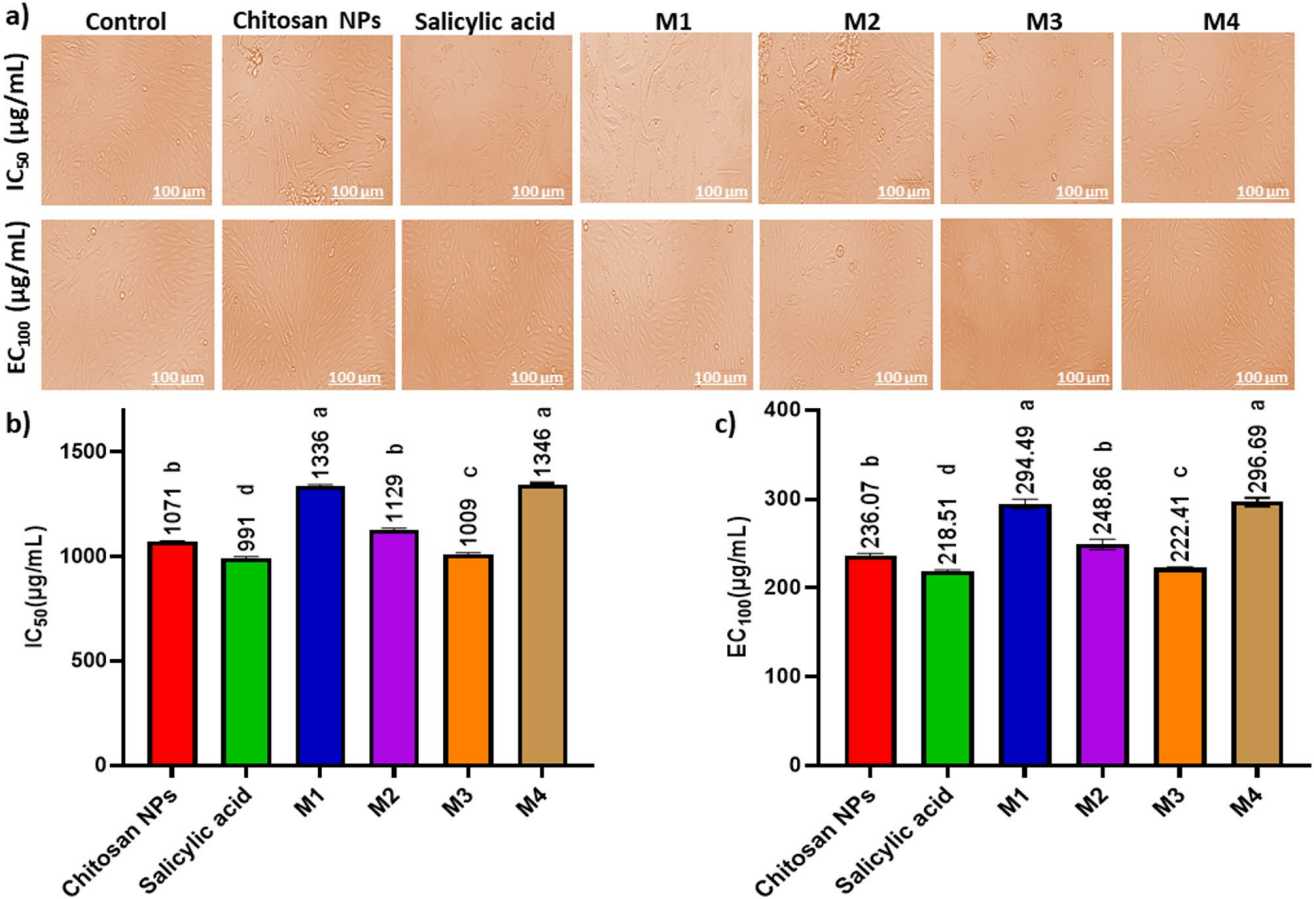
Effect of the safe dose (EC_100_) and 50% inhibitory concentrations (IC_50_) of the tested compounds on human skin fibroblast cells (HSF). (**a**) Morphological changes captured using a phase-contrast microscope. (**b**) IC_50_ values (the concentration at which 50% inhibition occurs) (µg/mL) of the tested compounds (**c**) EC_100_ values (the concentration at which 100% effect is observed) (µg/mL) of the tested compounds. Data are presented as mean ± standard deviation (SD). Different superscript letters indicate significant differences across rows (*p* < 0.05), using GraphPad Prism 8.

Figure 8; Table 2 indicates a dose-dependent cytotoxic effect of the tested formulations on HSF cells, where higher concentrations (1000 µg/mL) resulted in lower cell viability, while lower concentrations (≤ 31.5 µg/mL) showed minimal cytotoxicity. Figure 9a shows the morphological viability of HSF after treatment with the tested compounds. Figure 9b, c) illustrate the 100% safe concentration range, which spans from 218.51 to 296.69 µg/mL, with salicylic acid identified as the least safe compound. According to ISO 10993-5 biocompatibility guidelines, a material is considered non-cytotoxic if cell viability remains ≥ 70% compared to the negative control. This reinforces that our nanoformulation is suitable for biomedical applications, including wound healing and antibiofilm coatings, while maintaining both safety and efficacy. Additionally, the natural origin, biodegradability, and FDA-approved status of chitosan further support its medical applicability^65^. However, the nanoforms prepared using methods 1 and 4 (M1 and M4) demonstrated the highest EC_100_ values, making them the safest nanoparticles. The IC_50_ values of chitosan NPs fall within the published range of 20 to 2500 µg/mL^66,67^. Furthermore, the IC_50_ of the salicylic acid in this study was 991 µg/mL aligning with the reported IC_50_ at 828.72 µg/mL^68^.

**Table 2:** Cell viability percentage of human skin fibroblast cells (HSF) cells treated with tested compounds. Data are presented as mean of the cell viability percentage ± sd.

Concentration (µg/mL)	Chitosan-NPs		Salicylic- acid		M1		M2		M3		M4	
	% Cell viability	SD	% Cell viability	SD	% Cell viability	SD	% Cell viability	SD	% Cell viability	SD	% Cell viability	SD
1000	42.92	2.67	47.39	2.76	53.83	1.53	51.59	1.40	47.45	0.96	54.34	0.44
500	76.85	4.15	76.28	1.91	69.52	3.38	73.60	1.85	78.89	1.15	77.23	0.84
250	78.64	1.66	82.21	2.87	82.72	2.87	89.03	1.59	87.37	1.28	81.44	0.77
125	83.80	1.70	88.65	1.40	89.60	3.64	89.16	0.32	93.81	1.28	88.46	1.47
62.25	91.01	5.55	91.77	2.04	90.65	1.98	92.03	1.98	90.82	0.96	92.41	1.89
31.5	94.26	4.85	94.77	3.93	96.82	2.04	97.39	2.30	95.34	4.40	99.74	1.64

### Antibiofilm Inhibition assay

**Fig. 10:**
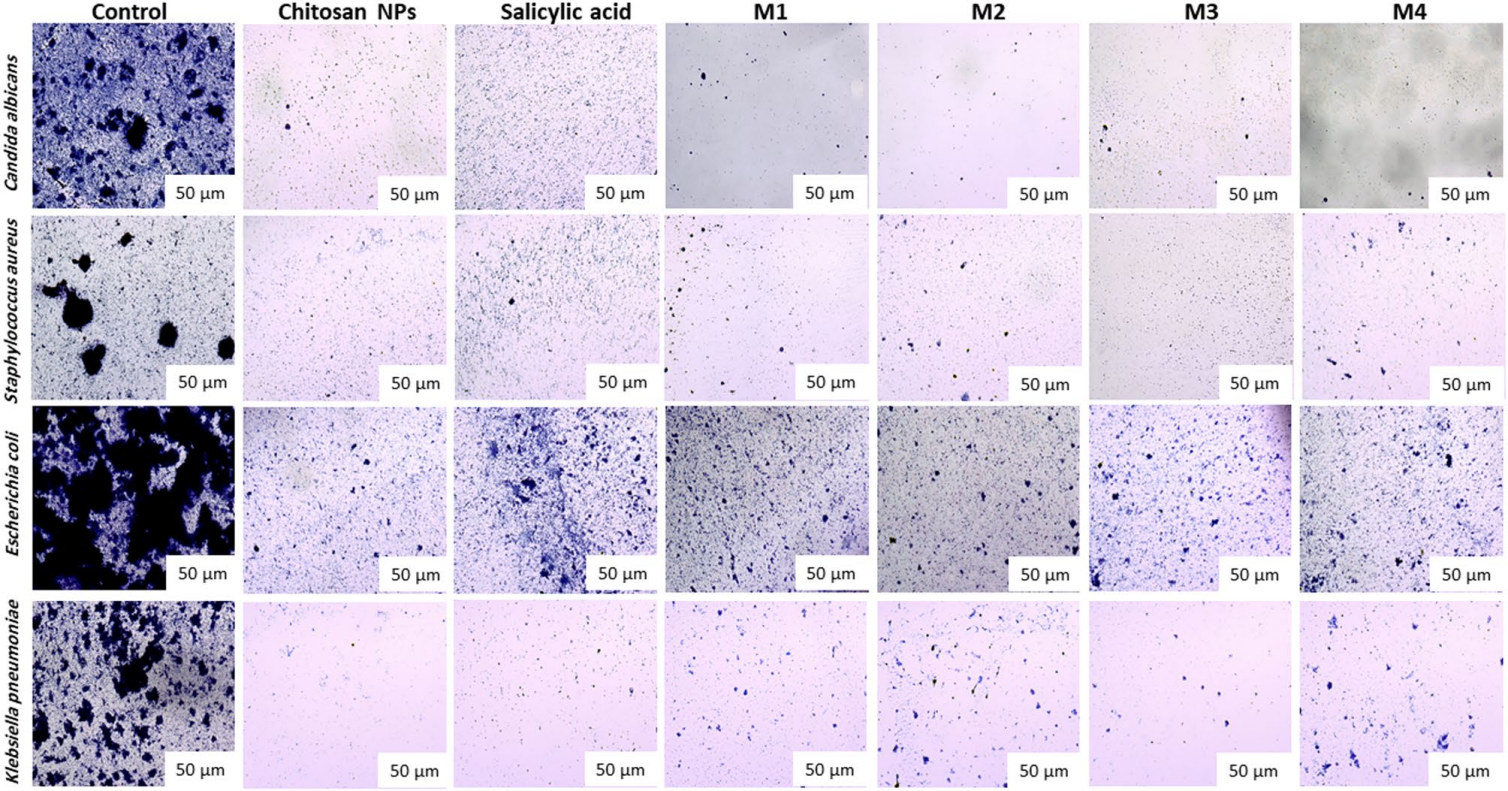
Light microscopy images showing the biofilm inhibition of the tested compounds against different microorganisms at the EC_100_ concentrations of the studied compounds.

**Fig. 11:**
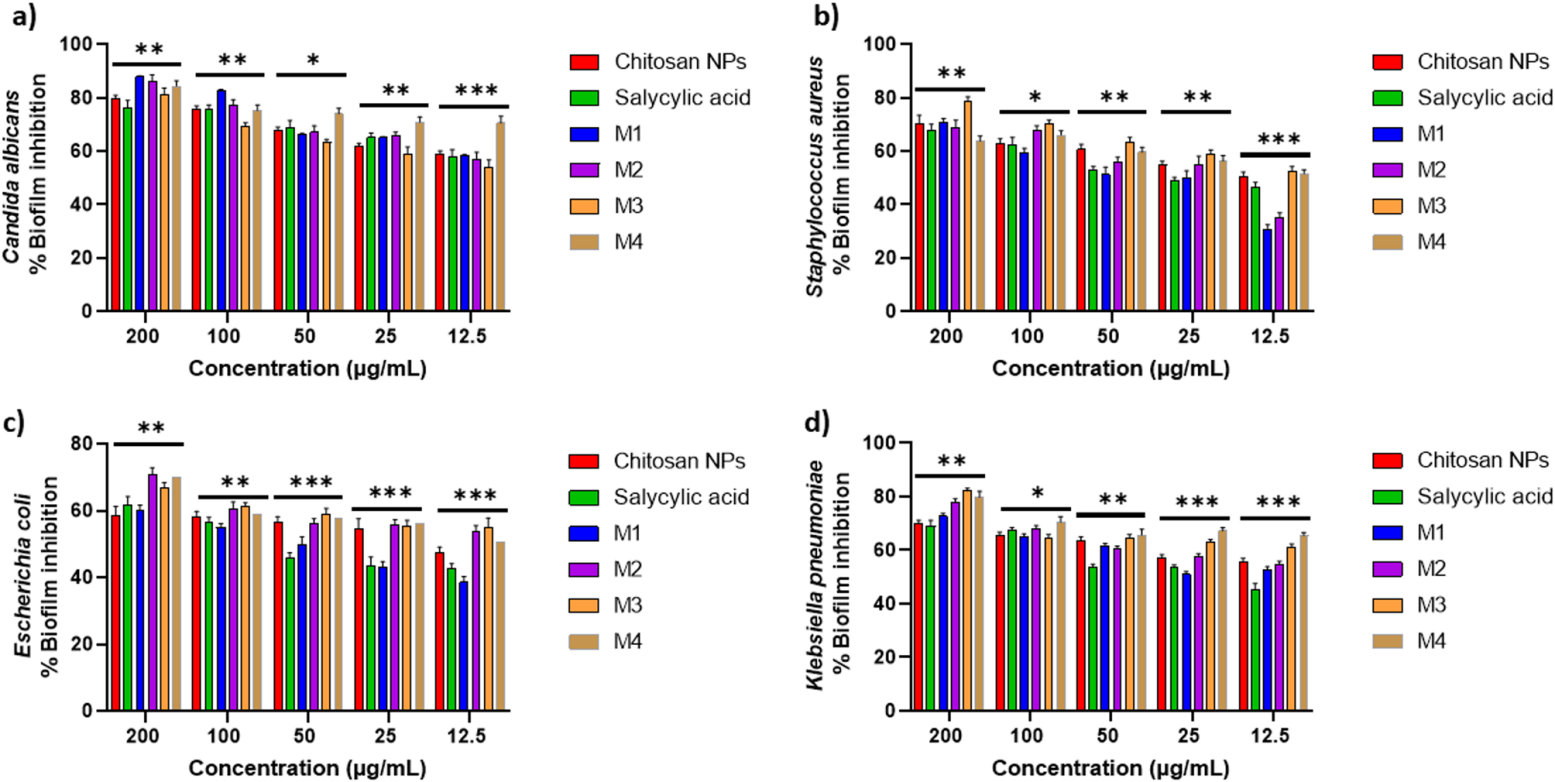
Biofilm inhibition percentage of different concentrations (200 –12.5 µg/mL) of the tested compounds against (**a**) *Candida albicans*; (**b**) *Staphylococcus aureus*; (**c**) *Escherichia coli*; (**d**) and *Klebsiella pneumoniae*. The bars on the graphs represent the mean ± SD of three independent replicates. The significant differences between groups: *p < 0.05, **p < 0.01, **p < 0.001.

The compounds exhibit a significant antibiofilm effect against the tested microorganisms at their safe concentrations. Figure 10 presents the qualitative morphological observations under a light microscope, illustrating the ability of the tested compounds to remove pre-formed biofilm after 24 h. Figure 11**(a–d)** presents quantitative data on the percentage inhibition of biofilm formation by the tested microorganisms: (a) *Candida albicans*, (b) *Staphylococcus aureus*, (c) *Escherichia coli*, and (d) *Klebsiella pneumoniae*. The nano-formulations demonstrated a higher percentage of inhibition, possibly due to their greater surface negativity. The nanoform combining chitosan and salicylic acid shows superior efficacy compared to either compound alone against almost all tested microorganisms. The percent inhibition of chitosan NPs and other nanoforms is dose-dependent and proportional to the concentration^69,70^.

Some studies have consistently attributed chitosan’s unique antibiofilm properties to its polycationic nature, which arises from the functional amino groups (NH₂) present in the N-acetylglucosamine units. Additionally, the polymeric structure of chitosan and chitosan oligosaccharides enables their chelation with essential metals such as calcium, zinc, and magnesium. These metals play crucial roles in bacterial gene transcription and translation; their sequestration disrupts these processes, ultimately leading to cell death^71^. Furthermore, chitosan NPs exhibit anti-quorum sensing (anti-QS) activity by interfering with bacterial cell-to-cell communication mechanisms or inhibiting the quorum sensing signalling pathways. This disruption prevents the formation of molecule-receptor complexes and the synthesis of various signalling molecules, effectively hindering biofilm development^72^. Additionally, chitosan has been utilized to safeguard medical devices, such as catheters and orthopedic implants, against infectious biofilm-forming pathogens. Chitosan coatings have also demonstrated the ability to eradicate substantial amounts of pre-existing viable biofilms of Staphylococcus epidermidis and *Staphylococcus aureus* on implant surfaces. This active protective effect of chitosan coatings on medical device surfaces represents a critical advancement in reducing the high risk of device-associated infections^71,73^.

Table 3 presents the fold reduction in biofilm formation. M1 achieved superior inhibition of *Candida albicans* biofilms (8.31-fold reduction), surpassing M2 (7.22-fold reduction). This performance aligns with findings by Gondim et al.^69^, emphasizing the role of polycationic nature in antibiofilm activity. M3 shows significant reduction against both *Staphylococcus aureus* and *Klebsiella pneumoniae*. Among the tested microorganisms, *Escherichia coli* exhibits the least fold reduction in biofilm formation, with M2 being the most effective formulation. Positively charged nanoparticles are more effective antibacterial agents due to their strong electrostatic attraction to negatively charged bacterial membranes, other factors such as shape and size also significantly influence their antibacterial efficacy^74^. Positively charged NPs enhance bacterial adhesion, disrupt cell structures through ion exchange, and promote reactive oxygen species (ROS) production, making them highly effective^75^.

**Table 3:** Fold reduction in biofilm formation of the tested compounds compared to the untreated control.

	Chitosan-NPs	Salicylic-acid	M1	M2	M3	M4
*Candida albicans*	4.95^e^ ± 0.029	4.21^e^ ± 0.091	8.31^a^ ± 0.088	7.22^b^ ± 0.042	5.38^d^ ± 0.033	6.42^c^ ± 0.067
*Staphylococcus aureus*	3.39^b^ ± 0.074	3.32^b^ ± 0.054	3.42^b^ ± 0.023	3.21^b^ ± 0.076	4.69^a^ ± 0.047	2.76^c^ ± 0.032
*Escherichia coli*	2.75^c^ ± 0.083	2.97^c^ ± 0.025	2.85^c^ ± 0.011	3.9^a^ ± 0.029	3.42^b^ ± 0.067	3.79^b^ ± 0.042
*Klebsiella pneumoniae*	3.34^d^ ± 0.036	3.22^d^ ± 0.035	3.66^c^ ± 0.089	4.54^b^ ± 0.052	5.56^a^ ± 0.028	4.95^b^ ± 0.017

Different superscript letters indicate significant differences across rows (*p* < 0.05).

Data are presented as mean ± standard deviation (SD).

The notable antibiofilm effect against *Candida albicans* (85% inhibition) can be attributed to chitosan’s ability to compromise fungal cell membranes, induce cytoplasmic leakage, sequester essential nutrients, and disrupt genetic processes. These mechanisms are influenced by factors such as molecular weight, degree of deacetylation, and the composition of the fungal cell membrane, particularly its unsaturated fatty acid content, which plays a role in determining susceptibility. Chitosan-based nanoparticles further amplify these effects due to their greater surface charge, expanded surface area, and enhanced cellular uptake, facilitating deeper biofilm infiltration and superior antifungal activity compared to conventional chitosan^12^. While chitosan’s antimicrobial efficacy varies among different microbial species—with some studies indicating higher effectiveness against Gram-negative bacteria^76^ and others reporting greater sensitivity in Gram-positive strains^77^—its strong inhibitory impact on *Candida albicans* in this study underscores its potential as a promising antifungal and antibiofilm agent.

## Conclusion

This study explored the synthesis of chitosan-salicylic acid nanoparticles using shrimp shell-derived chitosan, highlighting an environmentally sustainable approach. The nanoparticles were prepared using four different methods and thoroughly characterized to confirm their stability, morphology, and surface properties. FT-IR verified the successful conjugation of salicylic acid with chitosan, revealing characteristic functional group interactions. The FT-IR spectra showed peaks at 3450 cm⁻¹ corresponding to N-H and O-H stretching, with shifts in the amide peaks indicating interactions between chitosan and phosphate groups of TPP. The TEM and SEM analyses revealed morphological differences between formulations, confirming uniform particle sizes, spherical shapes, and variations in surface texture. The zeta potential measurements indicated positive values ranging from + 36.6 mV to + 41.3 mV for the different formulations, suggesting strong electrostatic stability and minimal aggregation. The antimicrobial and antibiofilm activities of the nanoparticles were evaluated against *Escherichia coli*,* Klebsiella pneumoniae*,* Staphylococcus aureus*,* and Candida albicans*, demonstrating significant biofilm inhibition, with suppression rates reaching up to 85% for *Candida albicans*. The nano formulation M4, prepared using sodium alginate, exhibited the highest antibiofilm efficacy, likely due to improved nanoparticle dispersion and controlled release of salicylic acid. The presence of sodium alginate as a crosslinking agent likely contributed to higher nanoparticle stability in aqueous environments, preventing rapid aggregation and ensuring a more sustained salicylic acid release for prolonged antimicrobial action. The cytotoxicity assessment confirmed the safety of the nanoparticles, with IC50 values ranging from 218.51 to 296.69 µg/mL, aligning with ISO 10993-5 biocompatibility guidelines. Among the tested formulations, M4 emerged as the most effective and sustainable approach. Sodium alginate improved nanoparticle stability in aqueous environments, preventing rapid aggregation and enabling a controlled release of salicylic acid for prolonged antimicrobial action. Compared to other methods, this formulation provided enhanced dispersibility and a more consistent particle size distribution. This method aligns with green chemistry principles by employing water as a solvent and using biodegradable, naturally derived polymers, making it a promising alternative for large-scale production.

These findings align strongly with the United Nations Sustainable Development Goals (SDGs), particularly SDG 3 (Good Health and Well-being) by addressing antimicrobial resistance, SDG 8 (Decent Work and Economic Growth) due to its potential to promote sustainable industries and innovations, SDG 12 (Responsible Consumption and Production) through the utilization of shrimp shell waste, and SDG 13 (Climate Action) by promoting eco-friendly synthesis methods. Moreover, the collaborative approach of this study supports SDG 17 (Partnerships for the Goals) by fostering multidisciplinary efforts to develop sustainable solutions. Future research should focus on optimizing the release kinetics, evaluating in vivo efficacy, and assessing the long-term stability of these nano-formulations to fully unlock their potential in healthcare and environmental settings.

### Limitations and future directions

While this study highlights the promising antibiofilm and antimicrobial properties of these nanoparticles, certain limitations must be acknowledged. The research primarily focused on biofilm prevention, without assessing the nanoparticles’ ability to disrupt pre-formed biofilms. Additionally, the impact of pH and ionic strength on nanoparticle stability and activity was not evaluated, which could influence their effectiveness in different environments. Moreover, the entrapment efficiency of salicylic acid within the nanoparticles was not determined, which is crucial for accurately interpreting the dosage and therapeutic potential. The role of these nanoparticles in quorum sensing inhibition, a key regulatory mechanism in biofilm formation, also remains unexplored. Furthermore, additional characterization techniques, such as X-ray diffraction (XRD), differential scanning calorimetry (DSC), and thermogravimetric analysis (TGA), could provide further insights into the crystalline structure, thermal stability, and degradation profile of the nanoparticles. To address these gaps, future research should investigate the nanoparticles’ ability to disrupt established biofilms and assess their activity under different environmental conditions. Further studies should explore their synergistic effects with conventional antibiotics to determine their potential as antibiotic adjuvants. Additionally, in vivo studies are necessary to confirm their therapeutic efficacy and biocompatibility, ensuring their suitability for clinical applications. Expanding the scope of analysis to include long-term stability studies and controlled release kinetics will provide deeper insights into their potential for real-world applications.

## Data Availability

The datasets generated or analysed during this study are accessible from the corresponding author upon reasonable request.
